# How Breakage of Bottles Can Be Reduced to a Minimum

**Published:** 1905-01

**Authors:** 


### HOW BREAKAGE OF BOTTLES CAN BE REDUCED TO A MINIMUM.*

The greatest obstacle that lies in the way of producing a sound container for liquids occluding gases under high pressure, as, for instance, solutions of hydrogen peroxide, is the fact that no process for making unbreakable glass has yet been discovered.

Up to the present the ordinary amber glass bottles have been found totally inadequate and untrustworthy, though a device patented by Mr. Charles Marchand goes far towards overcoming this delinquency.

This device practically reduces the danger of bursting of the bottles to a minimum. As long as the bottles having this device, are kept in stock standing up, the pressure resulting from shaking, high temperature in course of transit, etc., will not rise much above four or five pounds to the square inch; and, therefore, though occasionally a bottle may crack or burst, it is not due to pressure but to the inherent imperfection of the glass, arising either from the lack of homogeneity or else imperfect annealing, or both, to which we have already referred.

The worse feature of this unreliability in the bottle is, that there is no accurate way of detecting it. A bottle may be submitted to a pressure of a hundred pounds to the square inch without betraying signs of weakness, yet even with nothing in it it may burst or crack within an hour.

The only remedy in these conditions as to the bottles, and that is not absolute, is in changing the material from which the containers are made and substituting for the unreliable amber glass a good article of flint glass. While, as we have intimated, this does not absolutely remove the danger of loss by explosion or cracking, it greatly reduces it, and when the flint glass container is closed by Marchand's Safety-Valve Stopper, danger is reduced to a mini-

*Abstract from the *National Druggist*, of Saint Louis, Mo., October, 1904.

mum, beyond which in the present condition of the technics of bottle-making it is impossible to go.

This is exactly what Mr. Charles Marchand, the manufacturer of hydrozone, glycozone, peroxide of hydrogen, etc., intends to do. Just as soon as his present stock of amber glass containers is exhausted he will use exclusively flint glass, every bottle being corked with an automatic safety-valve stopper. By adopting these expedients, Mr. Marchand, having done all in his power to prevent breakage, can go only one step further—to make good any losses from that direction—replace the bottles that get broken from this cause. Beyond this it would be unreasonable to expect him to assume further responsibility. The actual danger to life or limb from the bursting of a bottle of hydrogen peroxide or any of Mr. Marchand's preparations, is trivial as compared with those arising from the explosion of bottles of beer, ginger ale, champagnes and other sparkling wines, or even Apollinaris or other heavily aerated waters.

When any of these rupture the fragments are driven not only with all the force and energy of the already liberated gases, but with the augmented energy of the residual gas suddenly set free, and so may inflict severe, sometimes irreparable damage. The safety-valve arrangement in the stopper of bottles of hydrozone prevents the sudden disengagement of a great volume of gas.

Assuming that through some imperfection of the stopper the puncture should close as soon as the pressure from within rose to a point far within that required for the rupture of the bottle, the stopper not being wired, but merely tied down, will be forced out.

But glass is a proverbially brittle and treacherous substance, and it is liable to break in the hands of anybody at any moment and without any discoverable or apparent cause, and that whether filled or not. As a consequence there must always be some risk attached to the handling of glass containers. The best that can be done, as we have suggested elsewhere, is to reduce the risk of rupture or fracture to a minimum, and this Mr. Marchand has done, not only by his safety-stopper device, but also by the promised substitution of the stronger flint glass. The retail trade will, we are sure, welcome this latter change most heartily, since it completes and supplements the efforts made in the mechanical direction, and thus removes, as far as lies in human efforts, all danger arising from handling Marchand's goods.

